# Diagnosis and Treatment of Dandy-Walker Syndrome With Two Types of Ventriculoperitoneal (VP) Shunts: A Case Report

**DOI:** 10.7759/cureus.46564

**Published:** 2023-10-06

**Authors:** Mohammed Khaleel I.KH. Almadhoun, Abdallah Wasel Hattab, Nemer Nedal Alazzeh, Sufyan Taleb Aladwan, Osamah Ta’amneh

**Affiliations:** 1 Medicine and Surgery, Mutah University, Al-Karak, JOR; 2 Neurosurgery, Mutah University, Al-Karak, JOR; 3 Neurosurgery, Thamar University, Amman, JOR; 4 Neurological Surgery, Hashemite University, Amman, JOR; 5 General Practice, National Center for Diabetes, Endocrinology and Genetic Diseases, Irbid, JOR

**Keywords:** pediatric hydrocephalus, congenital neurological condition, endoscopic third ventriculostomy, ventriculoperitoneal shunt, dandy-walker syndrome

## Abstract

Dandy-Walker Syndrome (DWS) is a rare congenital neurological condition characterized by cerebellar and posterior fossa malformations, often presenting a variable clinical spectrum. Common complications include hydrocephalus, necessitating interventions like ventriculoperitoneal (VP) shunts, and endoscopic third ventriculostomy (ETV). We describe the case of a five-month-old infant conceived through in vitro fertilization (IVF), initially presenting with cold-like symptoms, later diagnosed with DWS. The patient underwent VP shunt placement for hydrocephalus management, with subsequent complications requiring shunt revisions and ETV. Vigilant monitoring and timely interventions were crucial for a favorable outcome, highlighting the challenges in diagnosing and managing DWS and the importance of tailored treatment strategies.

## Introduction

Dandy-Walker Syndrome (DWS) is a rare congenital brain malformation primarily affecting the fourth ventricle and the cerebellum. Originally described by W. Dandy and K. Blackfan in 1914, it was officially designated as Dandy-Walker Syndrome in 1954 by C. Benda [1,2]. Benda's early work also drew attention to familial cases within this syndrome, emphasizing the significance of exploring both its genetic and clinical dimensions.

Children diagnosed with DWS often present with a diverse range of clinical manifestations [2]. These include the enlargement of the fourth ventricle, partial or complete absence of the cerebellar vermis (the region situated between the cerebral hemispheres), cyst formation within the posterior fossa, and delayed motor development during early infancy [3]. DWS can also present with hydrocephalus, seizures, and increased intracranial pressure, leading to symptoms like irritability, vomiting, and convulsions [2,3]. DWS may also involve cervical nerve dysfunction, abnormal breathing patterns, agenesis of the corpus callosum, and malformations affecting various body parts such as the face, limbs, digits, and heart [2,4].

DWS arises during embryonic development and is thought to result from various factors that affect the formation of the cerebellar hemispheres and the fourth ventricle [4]. Predisposing factors for DWS include gestational exposure to rubella during the first trimester, cytomegalovirus, toxoplasmosis, and exposure to substances like warfarin, alcohol, and isotretinoin [2-4]. Notably, there is a male-to-female ratio of 1:3 in some documented cases. DWS is a relatively rare condition, occurring in about one out of every 25,000 live births, with a higher prevalence in females than males [5,6]. The causes of DWS are complex, and familial cases have been well-documented, indicating a multifaceted etiology [2].

## Case presentation

A five-month-old male infant was admitted to the hospital on May 3, 2022, after presenting with symptoms resembling a common cold for two days such as nasal congestion, cough, and low-grade fever. The infant’s mother reported that he was born at term, with no complications during pregnancy or delivery, and that he had received all the recommended vaccinations. The infant had no known allergies, no previous hospitalizations, and no family history of neurological disorders.

Examination

**Figure 1:**
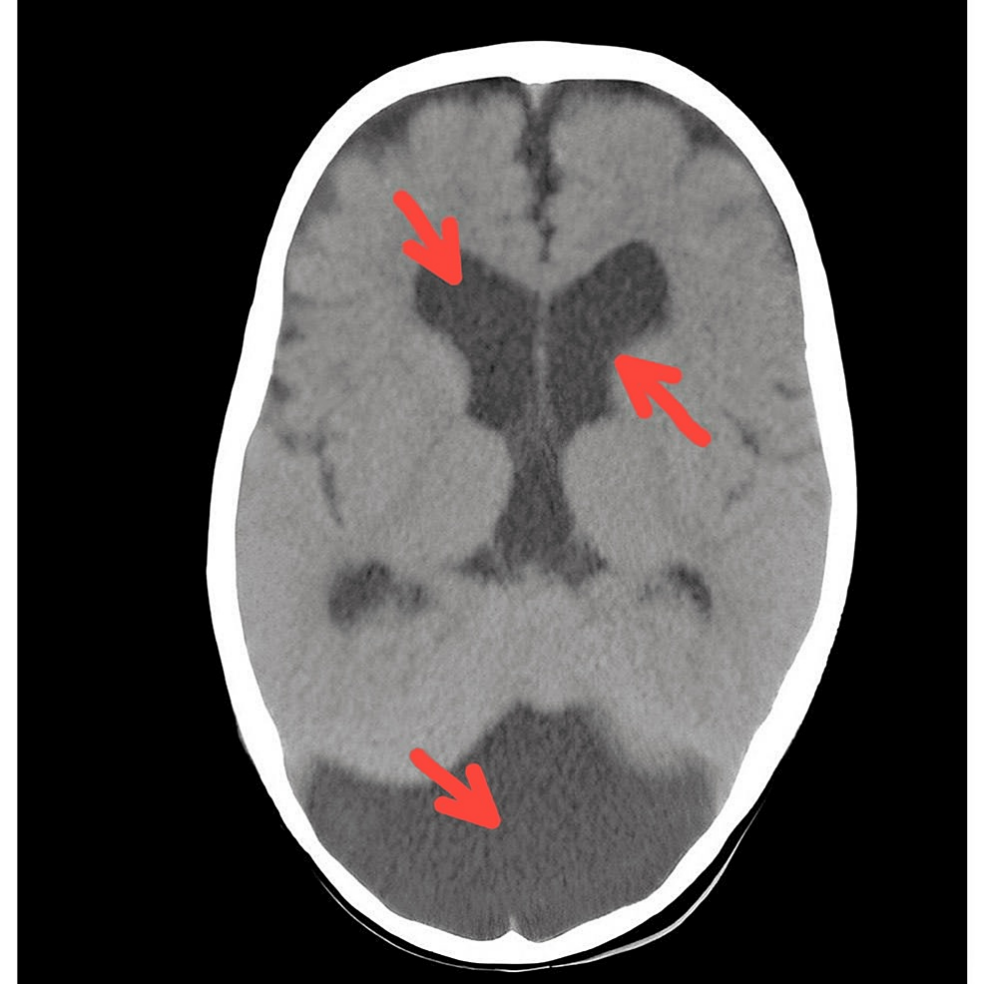
Brain CT scan finding; Enlarged posterior fossa with large cystic space continuous with the fourth ventricle associated with vermis and cerebellar hypoplasia.

The infant appeared alert and active, with normal skin color and hydration. His vital signs were temperature 37.8°C, pulse 120 beats per minute, respiratory rate 37 breaths per minute, blood pressure 82/50 mmHg, and oxygen saturation 98% on room air. His weight was 7.5 kg (50th percentile), his length was 66 cm (75th percentile), and his head circumference was 48 cm (above 97th percentile). The physical examination revealed no abnormalities, except for a slightly enlarged head, which was symmetrical and smooth, with no signs of increased intracranial pressure such as bulging fontanelles, sunken eyes, or irritability. The infant’s neurological examination was normal, with no focal deficits, seizures, or developmental delays. The infant’s chest, abdomen, and extremities were also normal, with no signs of infection, inflammation, or trauma. The infant’s laboratory tests, including complete blood count (CBC), electrolytes, liver and kidney function tests, and urine analysis, were all within normal limits. A cranial CT scan (Figure 1) was performed to further evaluate the infant’s enlarged head. The CT scan showed dilatation of the fourth ventricle with communication to a large posterior fossa cyst, accompanied by marked dilatation of both lateral ventricles.

**Figure 2:**
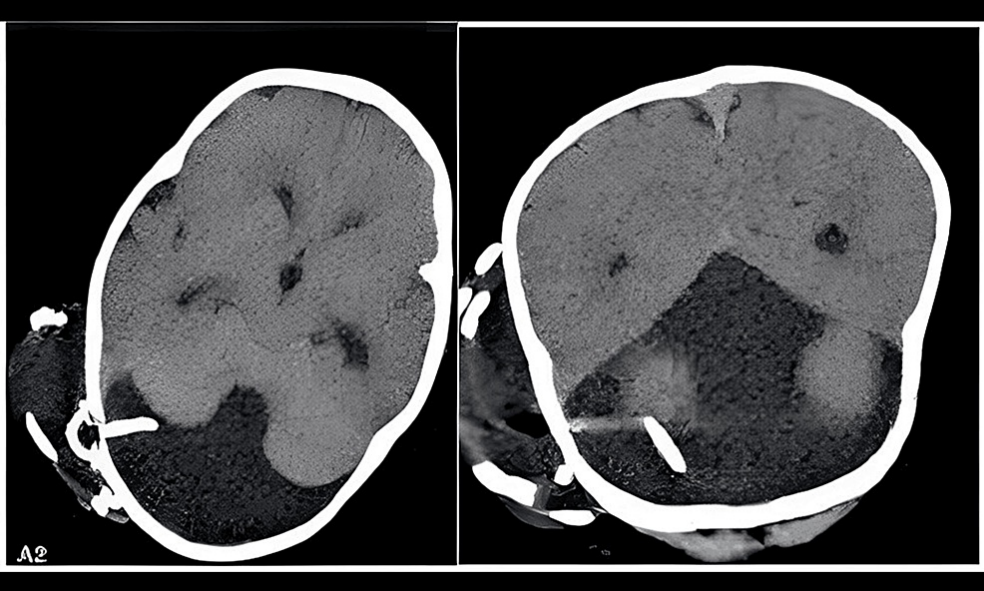
Brain CT scan finding; There is a right occipital approach.

Comprehensive discussions were held with the family, during which the nature of the condition and various treatment options were thoroughly explained. A consensus was ultimately reached to move forward with the placement of a ventriculoperitoneal (VP) shunt in the fourth ventricle (Figure 2). After the successful surgical procedure, the infant was discharged from the hospital following a five-day stay.

Approximately five months later, the same infant was readmitted to the hospital due to the abrupt onset of hypoactivity, sleepiness, and poor feeding. Subsequent to these symptoms, a repeat cranial CT scan was performed, revealing the enlargement of the ventricles and the displacement of the previously placed VP shunt. An urgent surgical intervention was carried out to reposition the VP shunt to its original position, resulting in a marked improvement in the child's overall condition. However, seven days post-surgery, the infant developed a fever, which prompted a comprehensive medical evaluation. Laboratory analyses, including (1) CBC, (2) C-reactive protein (CRP) assessment, and cerebrospinal fluid (CSF) analysis, collectively indicated the presence of an infection.

Based on the following results: (1) CBC results show elevated WBC count (result: 20.42 unit: 10^9/L; reference range: 6-11) and increased neutrophil percentage (41.1%). (2) CRP level (result: 50 unit: mg/L; reference range: less than 10). (3) CSF culture analysis with Gram stain results reveals the presence of coagulase-negative staphylococci (CNS), a bacterium commonly found on the skin. The antibiogram results indicate that the CNS is susceptible to penicillin, amoxicillin, amoxicillin/clavulanate, and flucloxacillin, while displaying resistance to fusidic acid.

**Figure 3:**
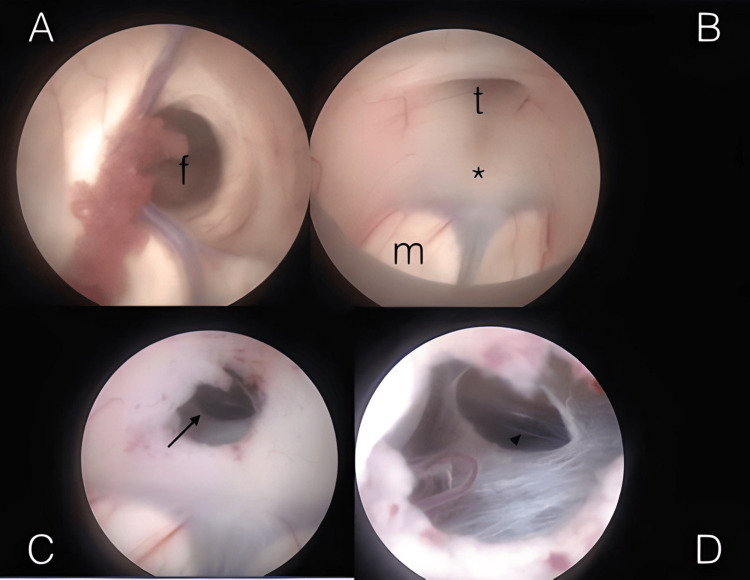
Endoscopic third ventriculostomy (ETV) (A) This endoscopic image displays the foramen of Monro (f) and its surrounding boundaries. (B) In this endoscopic view, you can observe the floor of the third ventricle before creating a perforation for ETV. The planned perforation site is marked with an asterisk (*) and is located between the tuber cinereum (t) and the mammillary bodies (m). (C) Following the perforation, this endoscopic image provides visualization of the Liliequist membrane, indicated by the arrow. (D) After opening the Liliequist membrane, this endoscopic view shows the basilar artery, as indicated by the arrowhead.

As a result, the medical team opted to remove the infected VP shunt and replace it with an external ventricular drain (EVD). Simultaneously, antibiotic therapy was initiated (250 milligrams (mg) of amoxicillin and 125 mg of clavulanate every 8 hours), which led to a gradual reduction in the fever over the span of one week. Subsequent CSF analysis revealed normal results. In line with the long-term management strategy, a plan was devised to perform endoscopic fenestration of the third ventricle (ETV success score 40%; five-month-old = 10%, aqueductal stenosis = 30%, previous shunt = 0%) (Figure 3). This procedure was smoothly executed, with careful and continuous monitoring of the infant's neurological status to ensure their well-being.

The only follow-up was after three months which showed normal physical examination, normal vital signs, normal head circumference (46.5 cm), and the child started to increase weight by 3.2 kg.

## Discussion

This case underscores the complexities inherent in diagnosing and managing DWS, especially in infants conceived through IVF. It highlights the critical importance of vigilant monitoring, timely surgical interventions, and involving families in the decision-making process to achieve positive outcomes. The multidisciplinary approach and adaptable strategies employed in this case illustrate the intricate nature of pediatric neurosurgical care.

When assessing the fetal brain, ultrasound serves as the primary imaging modality, offering an accurate evaluation of various aspects, including head circumference, bilateral thalami, lateral ventricles, choroid plexus structure, cavum septum pellucidum, cerebellum, cisterna magna dimensions, nuchal fold, and spine [7]. Moreover, ultrasound often provides insights into other CNS abnormalities commonly associated with Dandy-Walker malformation [8]. The routine assessment of cisterna magna size during prenatal brain ultrasonography can raise concerns about congenital posterior fossa anomalies.

However, beyond the 20th week of gestation, magnetic resonance imaging (MRI) of the brain surpasses ultrasound in its ability to diagnose CNS anomalies [9]. This comprehensive approach to diagnostic imaging underscores the significance of selecting the most appropriate method at different stages of fetal development.

Mega cisterna magna represents a potential differential diagnosis for Dandy-Walker malformation [10,11]. It is characterized by an enlarged posterior fossa despite a normal cerebellar size and is typically identified by the presence of an expanded fluid collection beneath and often behind the cerebellum. Compared to Dandy-Walker malformation or cerebellar vermis hypoplasia, developmental issues associated with mega cisterna magna tend to be less severe [10,11].

On the other hand, cerebellar vermis hypoplasia is defined by a smaller vermis without the pronounced upward rotation commonly observed in Dandy-Walker malformation [12]. This condition may also present with cystic enlargement of the fourth ventricle or an expansion of the posterior fossa, both features typical of Dandy-Walker malformation [10,11]. Although sometimes referred to as the "Dandy-Walker variant," this label can be somewhat misleading. Prenatal diagnosis can be performed using ultrasonography after the cerebellar vermis has completed its growth, typically around the 18th week of pregnancy. Confirmation can be obtained through CT scans, while a conclusive diagnosis requires karyotyping and postnatal imaging [8,13,14].

High intracranial pressure commonly manifests as a clinical characteristic in the majority of patients, mainly attributed to conditions such as hydrocephalus or the presence of a cyst in the posterior fossa. As a result, the central goal of treatment centers on the reduction of intracranial pressure, frequently achieved through surgical interventions. Surgical approaches may encompass the utilization of ventriculoperitoneal or cystoperitoneal shunts. In specific cases, endoscopic procedures, such as endoscopic third ventriculostomy (ETV), may also be contemplated as a potential treatment option.

## Conclusions

Dandy-Walker malformation is a rare form of brain malformation characterized by the absence or underdevelopment of the vermis, leading to the enlargement of ventricles and the potential development of hydrocephalus. While it can potentially be identified during prenatal screening, it can also be diagnosed postnatally through brain CT or MRI scans. The most effective treatment to alleviate symptoms and prevent further neurological complications involves the placement of VP and cystoperitoneal shunts. This case underscores the critical importance of early detection of Dandy-Walker malformation and the prompt initiation of surgical intervention to manage intracranial pressure and associated neurological challenges.
